# The Analysis of Polyethylene Hip Joint Endoprostheses Strength Parameters Changes after Use inside the Human Body

**DOI:** 10.3390/ma14227091

**Published:** 2021-11-22

**Authors:** Arkadiusz Szarek, Przemysław Postawa, Tomasz Stachowiak, Piotr Paszta, Joanna Redutko, Katarzyna Mordal, Aleksandra Kalwik, Justyna Łukomska-Szarek, Marek Gzik, Kamil Joszko, Dariusz Rydz, Małgorzata Łągiewka, Bożena Gzik-Zroska

**Affiliations:** 1 Department of Technology and Automation, Faculty of Mechanical Engineering and Computer Science, Czestochowa University of Technology, 42-201 Czestochowa, Poland; postawa@ipp.pcz.pl (P.P.); tomasz.stachowiak@pcz.pl (T.S.); paszta@itm.pcz.pl (P.P.); jredutko@iop.pcz.pl (J.R.); kmordal@iop.pcz.pl (K.M.); aleksandra.kalwik@pcz.pl (A.K.); 2 Faculty of Management, Czestochowa University of Technology, 42-201 Czestochowa, Poland; j.lukomska-szarek@pcz.pl; 3 Department of Biomechatronics, Faculty of Biomedical Engineering, Silesian University of Technology, 41-800 Zabrze, Poland; marek.gzik@polsl.pl (M.G.); kamil.joszko@polsl.pl (K.J.); 4 Faculty of Production Engineering and Materials Technology, Czestochowa University of Technology, 42-201 Czestochowa, Poland; dariusz.rydz@pcz.pl (D.R.); malgorzata.lagiewka@pcz.pl (M.Ł.); 5 Department of Biomaterials and Medical Devices Engineering, Faculty of Biomedical Engineering, Silesian University of Technology, 41-800 Zabrze, Poland; bozena.gzik-zroska@polsl.pl

**Keywords:** hip joint, UHMW-PE, acetabular cup

## Abstract

The influence of dynamic loads resulting from human motor activity and electrocorrosion inside the human body on the strength parameters of artificial joint elements has not yet been investigated. Hip joint arthroplasty is the most common surgical procedure in the world that allows doctors to remove pain and restore motor skills in people with severe hip diseases, after accidents, and in the elderly. Based on the reports, this article assesses changes in the number of implanted endoprostheses in the years 2005–2019 and determines the trends and estimated changes in the number of implanted hip prostheses in the following decades. The study assesses changes in selected strength parameters of UHMW-PE polyethylene inserts of hip joint endoprostheses during their use in the human body. The research was carried out on appropriately collected samples from UHMW-PE cups removed from the human body with a known history and lifetime from 4 to 10 years. Patients’ body weight ranged from 735 [N] to 820 [N], and the declared physical activity was similar in the entire research group. As part of the research, the values of changes in dynamic modules and the mechanical loss coefficient were determined in relation to the share of the crystalline and amorphous phases of artificial UHMW-PE cups, removed from the human body after different periods of exploitation under similar operating conditions. The analysis of selected strength parameters was performed at a temperature of 40 °C, which corresponds to the working conditions inside the human body. On the basis of numerical studies, the influence of changes in material parameters on the deformation of the artificial acetabulum during the patient’s motor activity, which is one of the causes of fatigue destruction, was determined.

## 1. Introduction

Bone and joint dysfunctions are the primary cause of social and work absence of people all over the world. Osteoarthritis (OA) affects over 30% of the general population over the age of 30 and over 65% over the age of 65 [1,2]. Joint arthroplasty is an expensive procedure, depending on the area of operation, age and weight of the patient, the extent of the procedure, type of prosthesis and components used, however in most cases it is the only way to effectively and completely cure disability in millions of people around the world [3,4,5]. Despite the dynamic development in the field of materials engineering, it was not possible to develop an artificial friction node that would be universal and sufficiently failure-free to be implanted in all patients. Research is constantly being carried out on the introduction of modern materials for the components of artificial joints and the improvement of the lifetime of endoprostheses, with particular emphasis on the friction node [6,7]. The second main current of engineering research is the analysis of the state of stresses and strains in the elements of artificial joints and their impact on tissues [8,9]. Along with the progression of implanted endoprostheses, the number of reimplantation procedures increases, the cost and the social effects of which are diametrically higher than those of primary procedures. Revision arthroplasty requires the removal of the dysfunctional artificial joint and its replacement with a new one [10]. The procedure is many times more expensive and much more complicated than primary arthroplasty, more extensive, more burdensome for the patient, and carries a greater risk of failure and complications. Microbial infections after primary arthroplasty are about 1%, while after reimplantation they account for about 4% of procedures [11,12]. One of the main reasons for the necessity to conduct reimplantation procedures is the mechanical destruction of the elements of the artificial bio-bearing. For various combinations of friction pairs used in endoprostheses, the elements made of UHMW-PE polyethylene are most frequently damaged [13,14].

However, UHMW-PE polyethylene cannot be replaced due to its unique strength parameters, unavailable for other material groups, e.g., vibration damping, low frictional resistance, high hardness, good chemical resistance and excellent biocompatibility. An additional advantage of this type of inserts is their low price. However, UHMW-PE cooperating in artificial hip joints is characterised by the highest wear, which is in high degree due to the change in the internal structure of PE, i.e., the shape and size of polymer particles [15,16]. Despite the great development in the field of improving the strength parameters of polymeric materials used in orthopedics, the UHMW-PE acetabulum is still the weakest element, being the most common cause of damage or destruction of artificial joints. Understanding the process of destruction of UHMW-PE exploited inside a human joint may bring relatively large benefits and will allow to optimise the group of patients, in whom implantation of polyethylene inserts will allow for their long and trouble-free usage. Systematising changes in the mechanical parameters of UHMW-PE inserts and the correct selection of endoprosthesis elements in terms of goniometric and anthropometric analysis for a specific patient may significantly influence the extension of the lifetime of polyethylene hip cups inside the human body.

## 2. Statistical Evaluation of the Number of Surgeries and Costs of Hip-Joint Replacement in Poland

**Figure 1:**
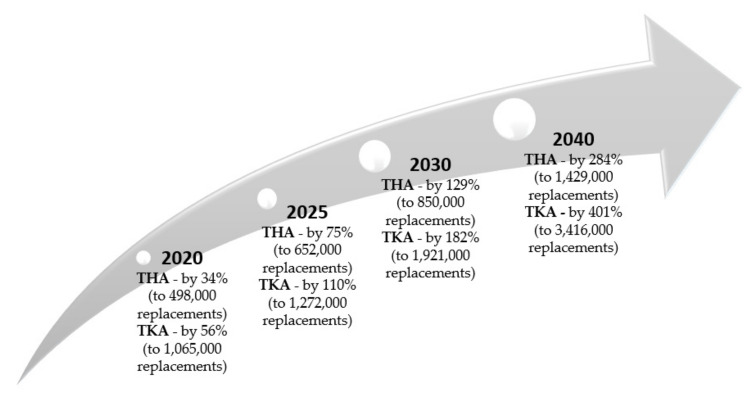
Growth forecast for total hip arthroplasty (THA) and total knee arthroplasty (TKA) in 2020–2040 (author’s own study based on [18]).

All over the world, the number of arthroplasty, mainly total hip arthroplasty (THA) and total knee arthroplasty (TKA) procedures is growing rapidly [17], which generate significantly increasing costs and often worse clinical results related to revision surgery. The forecasts for 2020–2040 confirm the progression of the number of arthroplasties for THA from 34% in 2020 to 284% in 2040, and for TKA from 56% to 401% [18]. A detailed summary in this regard is illustrated in Figure 1 below. The escalation of performed arthroplasty procedures has also been observed in recent years in Poland [19]. In 2019, a total of 94,000 were carried out (5821 more compared to the previous year, which was 88,179).

**Figure 2:**
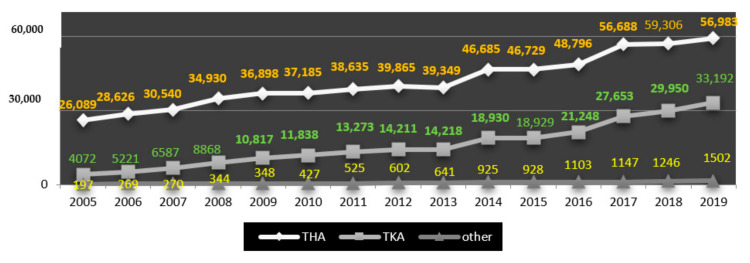
Number of individual types of joint replacement surgeries performed in Poland in 2005–2019 (author’s own study based on [19,20,21,22,23,24,25,26,27]).

To compare the scale of the phenomenon, in 2005 the annual number of articular arthroplasty was at the level of 30,378, so in relation to 2019 there was an increase by 32.32% to 94,000, of which 59,306 concerned the hip joint (49,937 total prostheses, 8805 partial prostheses, 564 revisions without replacement of implant components), 33,192 the knee joint (30,615 total arthroplasty, 2403 partial arthroplasty, 174 revision procedures without replacement of implant components), 938 the shoulder joint and 245—the elbow joint [20,21,22,23,24,25,26,27]. Detailed data on the number of THAs and TKAs are shown in Figure 2.

**Figure 3:**
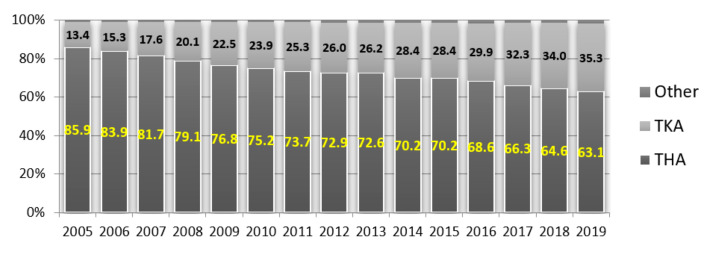
Percentage of individual types of joint replacement surgeries performed in Poland in 2005–2019 (author’s own study based on [19,20,21,22,23,24,25,26,27]).

The highest number was observed for hip joint replacement surgeries performed in 2005–2019, although with a steadily decreasing trend, amounting for 85.9% of all joint replacement surgeries in 2005, and 63.1% in 2019, which translated into a decrease of 22.8%. However, throughout the study, the escalation of knee joint replacement surgeries was observed as their percentage increased from 13.4% in 2005 to 35.3% in 2019, as shown in Figure 3 [19,20,21,22,23,24,25,26,27].

**Figure 4:**
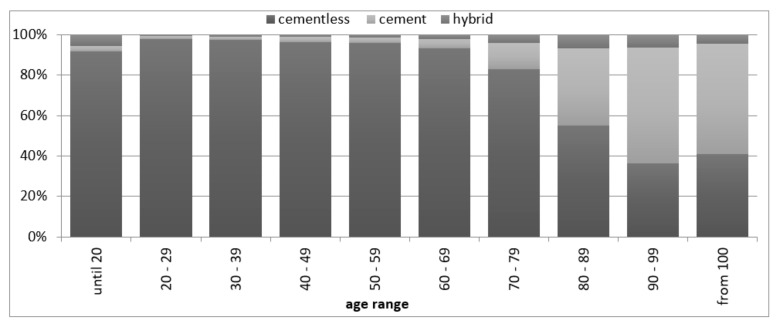
The percentage share of individual types of hip joint prostheses in age groups in 2019 (author’s own study based on [27]).

In 2019, 54,490 primary hip arthroplasty (91.88%) and 4816 (8.12%) revision arthroplasty procedures were performed (including 564 revision operations without replacing components). Among the prostheses implanted in 2019, cementless endoprostheses accounted for 83.63% (in 2005, about 46%), cement-based for 12.87%, and hybrid endoprostheses for 3.5%. The most uncemented endoprostheses were implanted in people between 60 and 79 years of age in 2019, while cemented ones between 70 and 89 years of age, which is illustrated by the data in Figure 4 [27].

**Figure 5:**
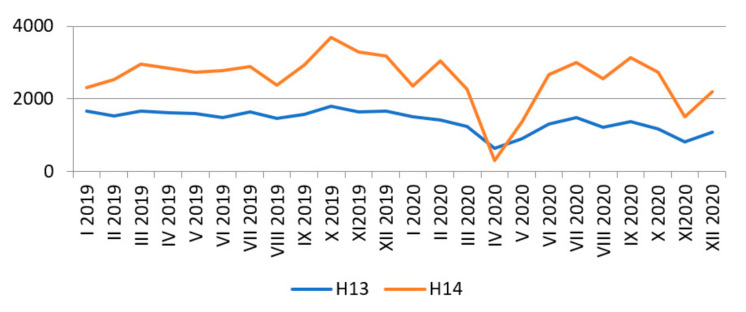
Comparison of the number of hospitalisations for hip replacement code H13 and H14 in 2019–2020 (author’s own study based on [28]).

Unfortunately, the COVID-19 pandemic, as indicated by the data of the National Health Fund in Poland, resulted in the reduction of hip replacement procedures, especially in the second quarter of 2020. A summary of the number of hospitalisations separately for: (1) code H13—primary total hip replacement and (2) code H14—primary total hip replacement with bone reconstruction, hip arthroplasty using a metaphysical steam and resurfacing replacement are illustrated in Figure 5 [28]. Based on the analysis of Figure 5, it can be noticed that from March 2020 the number of hospitalisations began to decrease, and in April the studied parameters reached the lowest values, then 925 hospitalisations of hip arthroplasty were settled (of which 626 were accounted for code H13, and 299 for H14). During this period in Poland, a lockdown was introduced due to the growing number of people infected with the SARS-CoV-2 coronavirus. At that time, the authorities decided to suspend most of the planned procedures, including arthroplasty.

**Figure 6:**
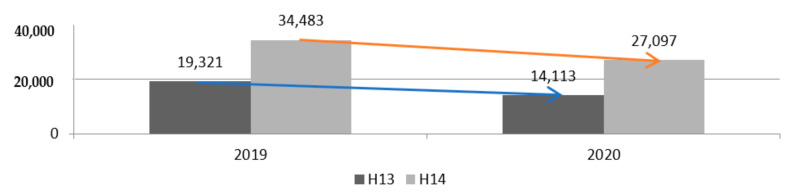
Total number of hospitalisations for hip replacement code H13 and H14 in 2019–2020 (author’s own study based on [28]).

The same situation happened during the second wave of the intense spread of the virus in October and November 2020. The number of hospitalisations in November 2020 for the H13 code was 821, and 1514 for H14. The comparison of the total number of hospitalisations of hip arthroplasty in the years 2019–2020 is shown in Figure 6 [28].

When analysing Figure 6, it can be noticed that in the first seven months of 2020, the number of hospitalisations for both the analysed H13 and H14 codes decreased. In the case of code H13, the number of hospitalisations decreased from 19,321 to 14,113 (by 5208, approximately 27%) compared to 2019. However, in the case of H14, this decrease was at the level of approximately 21.4% from 34,483 in 2019 to 27,097 in 2020. Despite health care reforms in Poland, the waiting period for hip arthroplasty is long. The list of arthroplasty procedures broken down by provinces in Poland in 2020 is shown in Table 1. Among all arthroplasty procedures, the largest number, 10,599, were performed in the Masovian Voivodeship, and the lowest, 1715, in the Opole Province. Providers from the following Masovian Voivodeship, Greater Poland and Lesser Poland, performed a total of 25,400 endoprostheses, i.e., over 35% of all procedures.

**Figure 7:**
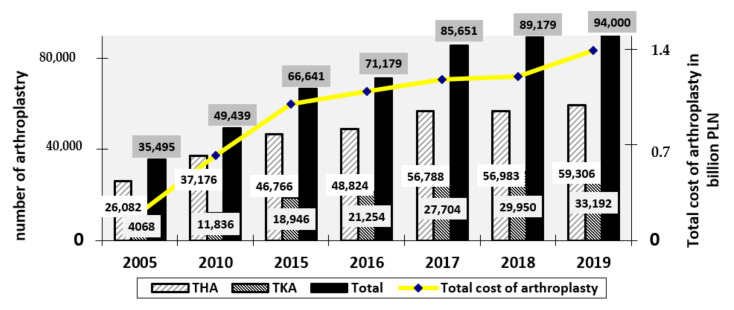
The number and costs of joint replacement surgeries performed in 2005–2018 (author’s own study based on [19,20,21,22,23,24,25,26,27]).

The costs of joint replacement surgeries in Poland were also growing, from 0.2 billion PLN in 2005 to over 1.39 billion PLN in 2019 (which translated into 0.77–5.92 billion EUR and 0.65–5.28 billion USD; using the mean exchange rate as of: 30 December 2005 of 1 EUR = 3.86 PLN and 1 USD = 3.26 PLN; 30 December 2019 of 1 EUR = 4.26 PLN and 1 USD = 3.80 PLN) [19,20,21,22,23,24,25,26,27,30,31]. Figure 7 presents the number and costs of joint replacement surgeries performed in 2005–2018. Cost reduction was observed in 2020, the cause of which was the stoppage of planned procedures due to the COVID-19 pandemic. From March 2020, the value of hospitalisation began to decline, and the studied parameters reached the lowest values. Their fall was observed again in October and November 2020 [29].

In summary, the statistical evaluation of arthroplasty in Poland shows that over the course of 15 years (2005–2019) both the number and costs of the performed procedures escalated, but the age structure of patients did not change significantly. The most numerous group of patients who underwent hip and knee arthroplasty are people aged 60–69 years, although an increasing number of implantations are also conducted in people aged 70–79. The share of cementless endoprostheses in the total number of hip arthroplasty benefits is growing. In 2005, uncemented endoprostheses accounted for less than 46% of all endoprostheses, and in 2019 almost 84%. The COVID-19 pandemic in 2020 forced the need to reduce the planned surgeries, the smallest number was carried out in April, October and November 2020.

## 3. Materials and Methods

### 3.1. Aim and Scope of Research

The main aim of the study was to assess the course of changes in selected strength parameters of polyethylene inserts used in the human body with a known history of exploitation and their impact on the strains state of the artificial joint acetabulum made of UHMW-PE. The choice of the research scope and goal was determined by the problem defined on the basis of national and international reports, resulting from a large percentage of operational damage of polyethylene hip joint inserts. Hip arthroplasty is the most common joint reconstruction procedure in the world, therefore extending the life of the prosthesis is a very important factor. The goal was achieved thanks to experimental tests, allowing us to determine the course of changes in selected mechanical parameters of polyethylene, and numerical tests, enabling the assessment of the state of strains of the cups during a known life cycle.

The null hypothesis H1 was adopted:

**Hypothesis** **1** (**H1**)**.** *As a result of the usage of polyethylene cups inside the human body, it does not change the strength parameters of the material and wear processes*.

And the alternative hypothesis H2:

**Hypothesis** **2** (**H2**)**.** *The period and intensity of using polyethylene cups inside the human body affects the changes in the strength parameters of UHMW-PE*.

The verification of the above research hypotheses has been carried out on the basis of experimental and numerical research.

### 3.2. Methodology of Experimental Research

The differential scanning calorimetry method (DSC) was used to determine the degree of crystallinity. The principle of calorimeters is to measure the difference in heat needed to keep the test sample and the neutral reference sample at the same temperature. Typically heating or cooling is programmed within a given temperature range and at a specific rate. The area between the peak of the DSC curve and the zero line is proportional to the change in enthalpy of the test sample. Ideally, the zero line should be parallel to the time axis (Q = O). In practice, it is usually deflected up or down due to changes in the specific heat (or mass) of the sample during the transformations.

The DSC method is used in research of:

- phase transitions of polymers;
- physical changes: glass transition temperature T_g_, melting point temperature T_m_;
- composition of mixtures and copolymers;
- thermal decomposition of Td (destruction temperature) of polymers, oxidation and combustion;
- heat of: crystallisation, polymerisation, dissolution, absorption and desorption;
- enthalpy of melting and degree of crystallinity.

Chemical reactions or transformations in which heat is released or absorbed appear on the DSC curve with a peak up or down from the baseline. Downward peaks correspond to exothermic processes (e.g., crystallisation) and upwards endothermic (e.g., melting). The determination of the degree of crystallinity by the DSC method is one of the most frequently used experimental methods and is denoted by the symbol ω*_c,h_*. The curve obtained during the DSC test is described by the following relationship:

(1) $$\frac{dQ}{dt}=m\cdot c_{p}\cdot \frac{dT}{dt}$$

where:

m—sample mass;
$c_{p}$—specific heat at constant pressure (heat capacity per unit mass);
$\frac{dQ}{dt}$—heat supplied or discharged to the system per unit time;
$\frac{dT}{dt}$—heating or cooling speed.

The DSC Phox 204 PC differential scanning calorimeter from NETZSCH (Selb, Germany), with measuring possibilities in the temperature range −190 °C to 600 °C with a heating rate from 0.1 to 50 °C/min, was used for the tests. The sample weights were determined with an accuracy of 0.001 g using a SARTORIUS microanalytical balance (SARTORIUS AG, Göttingen, Germany). The dynamic tests were performed with the use of the Netzsch DMA 242C device (NETZSCH, Selb, Germany). The FM-700 microhardness tester (FUTURE-TECH CORP., Kawasaki, Japan) and the FutureTech FV-700 hardness tester (FUTURE-TECH CORP., Kawasaki, Japan) were used to measure the hardness. Microscopic examinations and surface topography was assessed using a Keyence VHX-7000 digital microscope (KEYENCE INTERNATIONAL, Osaka, Japan).

### 3.3. Materials and Their Preparation

The research was carried out on appropriately collected samples from new UHMW-PE cups and those removed from the human body with a known history and exploitation time from 4 to 10 years. Patient weight within a known range was chosen as the main factor for the similar working conditions, as it is difficult to accurately assess the intensity of the load on individual samples over such a long period of time. Patients’ body weight (BW) ranged from BW_1_ = 735 [N] do BW_2_ = 820 [N]. The declared physical activity was similar in the entire research group.

**Figure 8:**
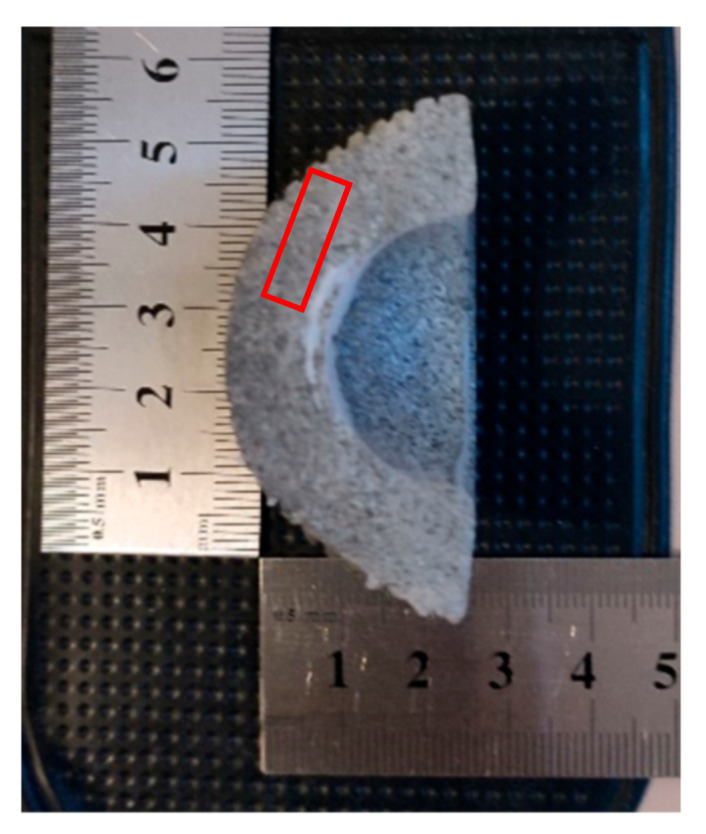
Preparation samples to tests.

The test samples were collected from the surface cooperating in the artificial joint with the metal head, from the areas with the highest degree of degradation. For structural tests using the DSC method, samples were taken from the surface to a depth of about 2 mm while maintaining the sample weight of about 10 mg. For the DMTA tests, samples were also taken from the work surface, but due to the method, the cut sample had to be formed into a rectangular beam with dimensions of 12 mm in length and a cross-section of 6 by 2 mm (Figure 8). Such treatment resulted in the destruction of part of the surface, but took into account the changes in the stiffness of the tested part.

In addition, for the analysed samples, the friction coefficient was also determined in the friction node in accordance with the ASTM D1894 standard, as well as the courses of changes in the dynamic modules and the mechanical loss factor. The results obtained from DMTA and DSC measurements allow the assessment of changes occurring in the PE cups during exploitation in the human body. Systematisation and determination of these changes will allow us to establish the relationship between: the influence of cyclic dynamic loads on the change of the material’s crystallinity degree, its molecular structure, and mechanical properties directly affecting the change of working parameters and changes of the E′ storage modulus and the tgδ coefficient of mechanical loss.

## 4. Results

### 4.1. Results of Numerical Research

**Figure 9:**
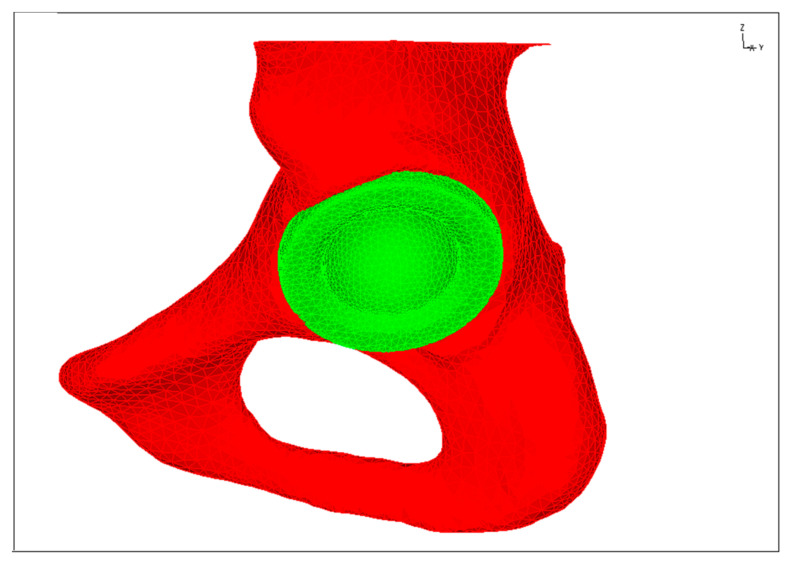
Numerical model of the pelvic bone after implantation of an artificial hip cup.

The assessment of the strains state of artificial hip joint cups was performed with the use of FEM numerical tools. The shape and size of the pelvic bone along with the implanted prosthesis was developed on the basis of computed tomography of a specific patient (Figure 9).

**Figure 10:**
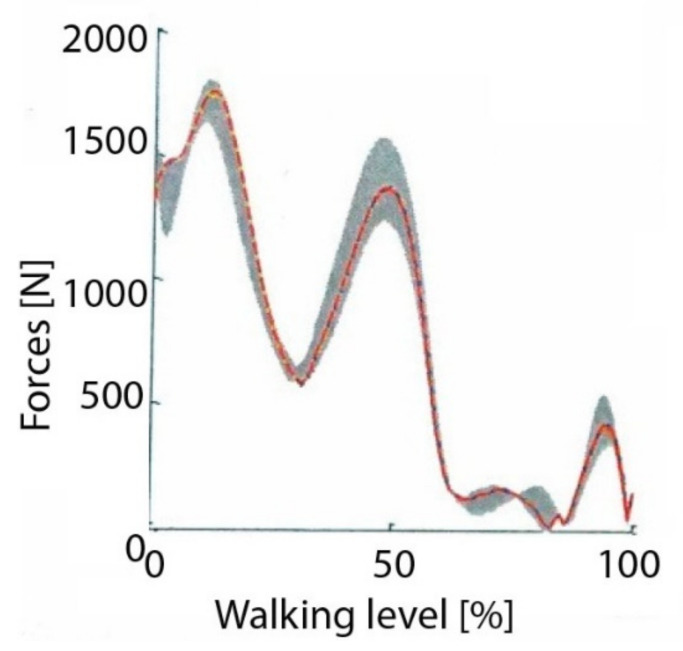
Values of forces loading the head-acetabular system during human walk [32,33].

The load on the hip joint was based on the dynamic model and the load on the limb during the gait phase. Two load variants were analysed for the extreme values of the body weight of patients who underwent reimplantation of the damaged artificial joint, i.e., BW_1_ = 735 [N] oraz BW_2_ = 820 [N]. The values of forces acting in the hip joint during the phase of walking at a speed of 4 [km/h] were developed on the basis of literature data and their characteristics are shown in Figure 10 [32,33].

The values of the forces loading the artificial cup during the maximum limb load in the gait phase for the patient’s weight BW1 = 735 [N] were: P1x = 228 [N], P1y = 419 [N] and P1z = −1646 [N], while for the patient with BW2 = 820 [N], the values of the forces were: P2x = 254 [N], P2y = 467 [N] and P2z = −1837 [N].

The cooperating head and cup materials were modeled as an element with linear-elastic mechanical properties, isotropic, for which the friction coefficient was μ = 0.15 and the strength parameters assumed the following values:

CoCrMo head, for which the assumed Young’s modulus E = 2.0 × 105 [MPa], Poisson’s ratio ν = 0.3, cup—the strength parameters of UHMW-PE, depending on the period of use, changed in accordance with the values obtained in experimental tests (Table 2). Young’s modulus E determined by the method of dynamic mechanical thermal analysis is the complex modulus E*, described by the equation:

E* = E′ + E″(2)

It consists of the real part E′ (storage modulus), which follows the deformation phase, and the imaginary part E″ (loss modulus), which is offset by π/2 from the deformation. Often, instead of the loss modulus E″, the tangent of the mechanical loss angle tgδ is given, described by the equation:

tgδ = E″/E′(3)

### 4.2. Research Results

**Figure 11:**
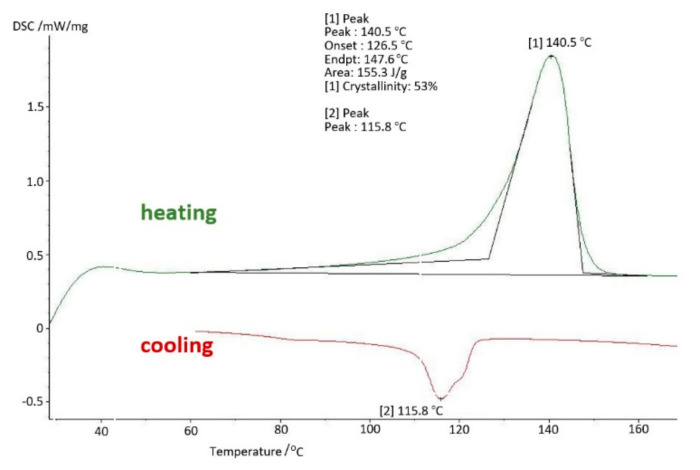
Differential scanning calorimetry (DSC) thermogram—base sample.

The results of tests using thermal analysis methods were presented in the form of changes in the values characteristic for a given method as a function of temperature change. Thus, for DSC (differential scanning calorimetry) studies, Figure 11 shows the changes in the DSC signal in mW/mg during heating (green line) and cooling (red line). As in the conducted research, it was important to know the properties of a specific sample and not the material itself, therefore the tests were limited to a single course of heating and cooling at a rate of 10 K/min. Subsequent heating would remove the thermal history of the test sample.

In the case of DMA tests, the following parameters were used:

- temperature range: RT up to 65 or 110 °C;
- heating rate: 2 K/min;
- strains amplitude: 120 µm;
- dynamic force: 5.2 [N];
- static force: 0.05 [N];
- frequency: 1 and 10 Hz.

**Figure 12:**
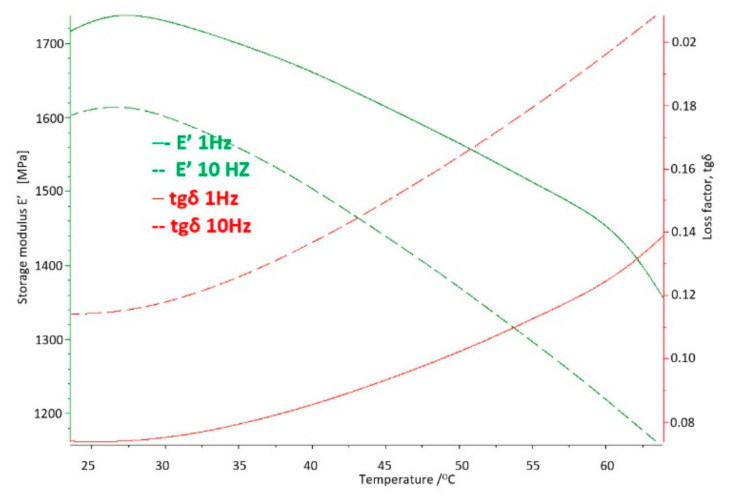
DMTA thermogram for the base sample made of UHMW-PE.

The basic sample was a new acetabulum, never used, not subjected to mechanical and chemical tests. The parameters obtained in the DMTA and DSC tests were the baseline measurements to which the results of the tests of samples used in the human body were compared. The test results for the base sample are shown in Figure 11 and Figure 12.

On the basis of the carried out research, it was determined that at the temperature of use of the artificial hip joint inside the human body, i.e., about 40 °C, with cyclical loads of 1 Hz close to the speed of the acetabular load during the patient’s gait, value of the storage modulus E′ = 528 [MPa], and mechanical loss coefficient tgδ = 0.13. The melting point of UHMW-PE was 140.8 °C and the enthalpy of fusion Q = 155.3 [J/g]. The degree of crystallinity of the base sample was K = 53%.

**Figure 13:**
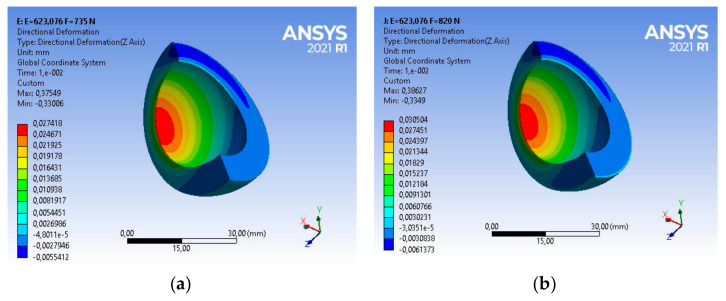
Strains distribution of the base acetabular during patient usage: (**a**) BW_1_ = 735 [N], (**b**) BW_2_ = 820 [N].

Taking into account the above strength parameters, the state of strains of the base acetabulum was assessed during the gait of patients with the assumed body weight. For a patient with a body weight of BW_1_ = 735 [N], the main deformations in the direction of the dominant vertical axis are equal to ε_z1_ = 0.37549, as shown in Figure 13a.

The use of this type of acetabulum in a patient with BW_2_ = 820 [N] causes a change in the strains state of the acetabulum (Figure 13b). Very good UHMW-PE parameters, i.e., vibration damping or low frictional resistance, will allow for a comfortable course of usage. The main strain in the direction of the dominant vertical axis is in this case ε_z2_ = 0.38627, and is greater by about 2% than the strain of the acetabular under the load BW_1_ = 735 [N], as shown in Figure 13a.

**Figure 14:**
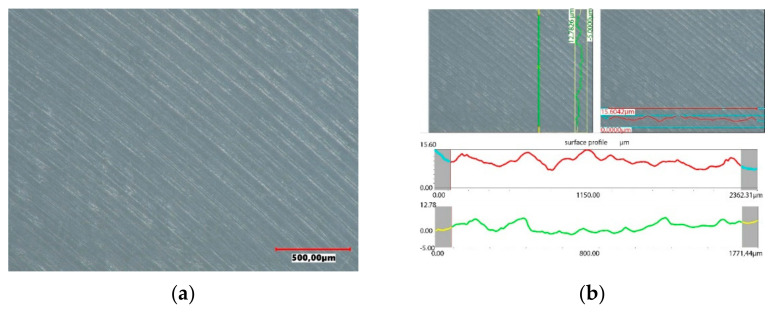
Surface profile of the base acetabular: (**a**) surface of the cup, (**b**) surface topography.

The accuracy of the new polyethylene hip joint cups is very high. The topography of the inner surface of the cup used as a friction node shows only slight traces of unevenness resulting from the machining of the surface, but their roughness does not exceed 15 µm. The surface topography was assessed using a Keyence VHX-7000 digital microscope. The image of the surface and the roughness profile of the new polyethylene insert is shown in Figure 14.

The evaluation of the strength parameters of UHMW-PE used in the human body for a very short time as an implant, i.e., 4 years, showed that the stiffness of the material increased. This effect is most likely the result of the influence of two factors: mechanical and chemical, and their interaction. During the use of polymer materials, their greater or lesser degradation is always dealt with. This is because the macromolecules that make up the polymers have a tendency to break under the influence of various factors, causing their length to become shorter and the molecular weight to decrease. However, also during the impact of high pressures, the processes of surface strengthening of the material and the associated increase in the degree of crystallinity may occur (it involves only partially crystalline materials).

**Figure 15:**
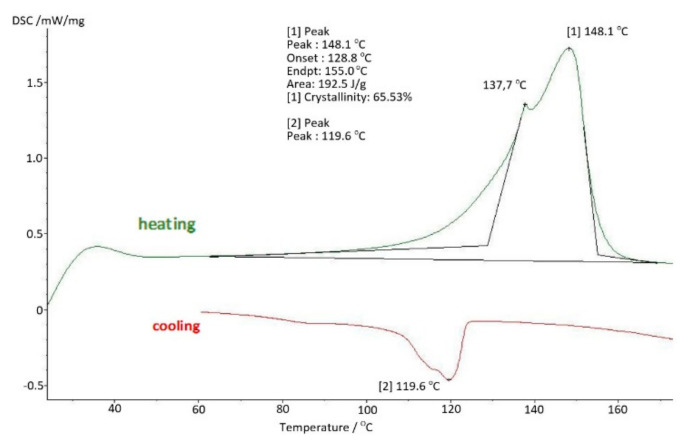
DSC thermogram: polyethylene used in the body for 4 years.

The use of UHMW-PE in the human body for 4 years causes changes in strength parameters and the degree of crystallinity. In comparison with the basic samples, a 23% increase in the degree of crystallinity of the samples was noted to the value of K = 65.53% (Figure 15).

**Figure 16:**
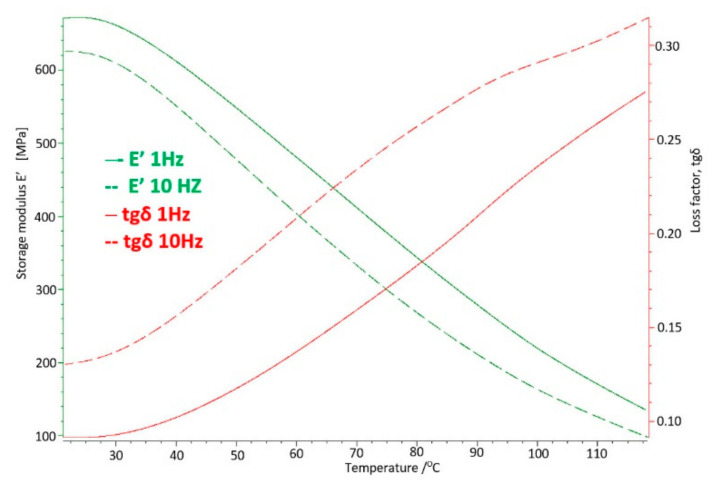
DMTA thermogram for polyethylene used in the body for 4 years.

Cyclic fatigue loads and the aggressive environment inside the human body for 4 years caused changes in the strength parameters of UHMW-PE and so for the storage modulus the value increased by about 5.5% to E′ = 557 [MPa], while the mechanical loss coefficient increased by about 23% to the value of tgδ = 0.16 (Figure 16).

**Figure 17:**
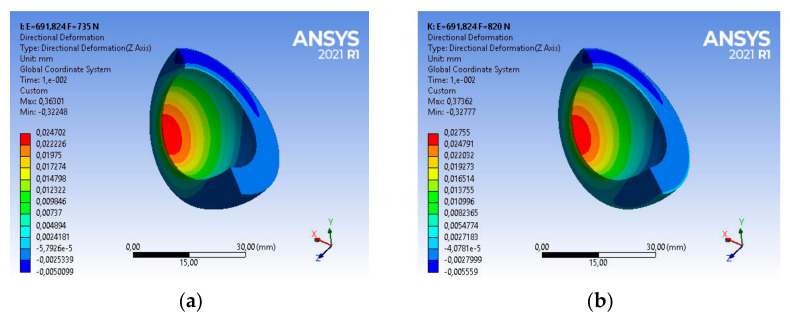
Strains distribution of the acetabular used in the body for 4 years during patient usage: (**a**) BW_1_ = 735 [N], (**b**) BW_2_ = 820 [N].

Taking into account the above strength parameters, the strains state of the acetabulum was assessed. For a patient with body weight BW_1_ = 735 [N], the main strain in the direction of the vertical axis are, respectively, ε_z1_ = 0.36301, while for a patient with BW_2_ = 820 [N], the value of strain ε_z2_ = 0.37362—in both cases it is about 97% of the base value. The nature and magnitude of the strains are shown in Figure 17.

**Figure 18:**
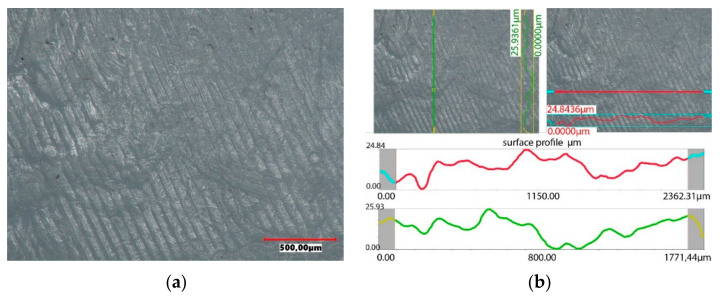
Surface profile of the acetabular used in the body for 4 years: (**a**) surface of the cup, (**b**) surface topography.

Under the influence of the loads acting on the endoprosthesis, a new accuracy of the contact surfaces is achieved on the surfaces of the artificial joint. The forces acting on the surfaces of the artificial joint are distributed only to the highest peaks of the roughness. Hence, the higher the precision workmanship, the more peaks come into contact with the cooperating joint components. The tops of rough surfaces deform elastically and after exceeding a certain pressure value in combination with frictional resistance, plastic deformation of the peaks occurs, connected with their micro-abrasion, as shown in Figure 18. Assessing the surface topography of the acetabulars used in the human body for 4 years, it was found that during this period the wear process is minimal. On the used surface, small scratches running in the direction of movement of the friction node were observed, however, from the operational point of view, this process can be treated as a matching of a pair of elements cooperating in an artificial bio-bearing. The size of surface roughness does not exceed 26 µm.

**Figure 19:**
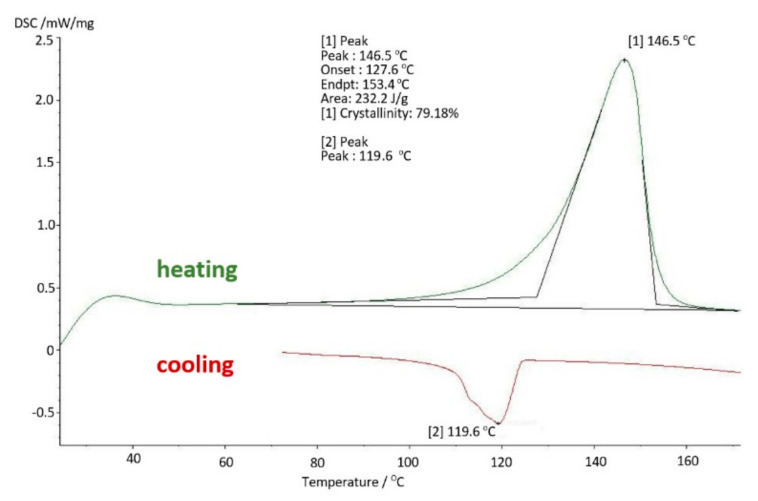
DSC thermogram for polyethylene used in the body for 8 years.

**Figure 20:**
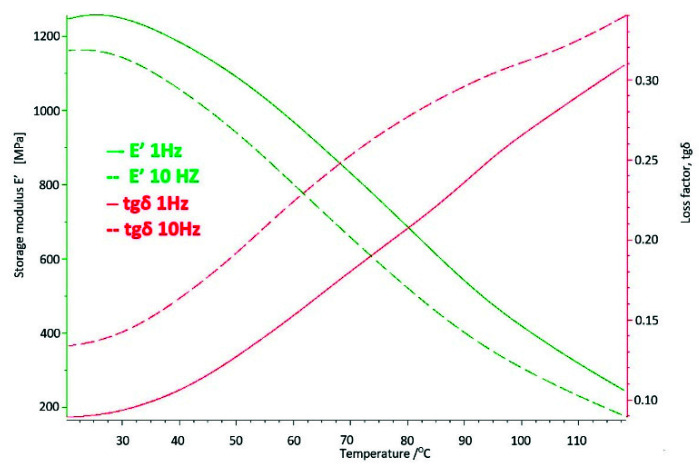
DMTA thermogram for polyethylene used in the body for 8 years.

With the extension of the period of use of UHMW-PE inside the body to 8 years, consecutive increase in the strength parameters of the material can be observed. This proves that cyclical dynamic loads resulting from the patient’s motor skills and the aggressive environment inside the body gradually degrade the polymer. The degree of crystallinity of the samples is K = 79.18%, which in comparison with the basic samples gives a nearly 50% increase in the proportion of the crystalline phase, and in comparison with the samples used for 4 years, this increase is about 20% (Figure 19). The analysed strength parameters of UHMW-PE take the following value: storage modulus E′ = 1164 [MPa] and the mechanical loss coefficient tgδ = 0.17. The melting point of polyethylene is 146.5 °C, while the enthalpy of melting T_m_ = 146.5 [J/g] (Figure 20).

**Figure 21:**
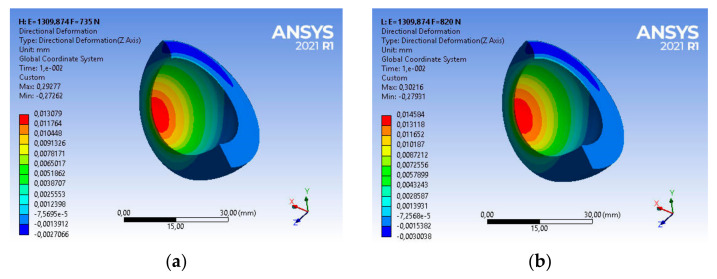
Strains distribution of the acetabular used in the body for 8 years during patient usage: (**a**) BW_1_ = 735 [N], (**b**) BW_2_ = 820 [N].

For such strength parameters during loads generated by the patient BW_1_ = 735 [N], the main strains UHMW-PE are equal to ε_z1_ = 0.29277 (Figure 21). Compared to the state of strains after 4 years of use, a nearly 20% decrease in strains was recorded.

**Figure 22:**
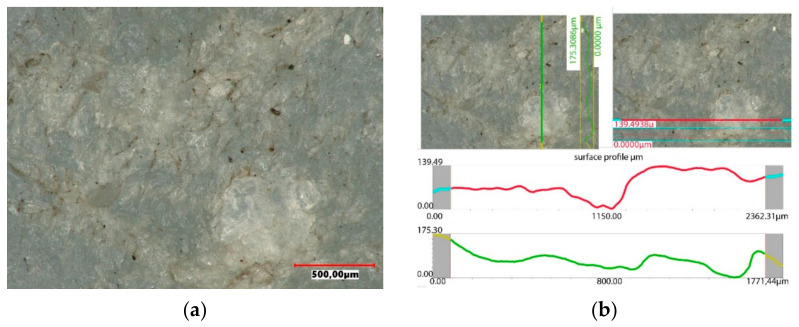
Surface profile of the acetabular used in the body for 8 years: (**a**) surface of the cup, (**b**) surface topography.

Over the operating time, the degradation of the surface progresses, which causes a change in the topography of the cooperating surfaces (Figure 22). The wear of the implant surfaces is primarily a set of processes taking place in the surface layer of the contact surfaces of the implants, characterised by the loss and displacement of mass and permanent deformation of the surface. In the 8-year period of use, high wear can be observed, characterised by quite deep scratches and the displacement of the UHMW-PE mass towards the main loads. The size of the resulting roughness is large and ranges from 130 µm to 175 µm. It can be assumed that the friction of the head on such a worn surface may generate micro-vibrations transferred to the acetabular and the frictional resistance is many times higher than in the new friction node of the artificial joint.

Analysing the strain state, taking into account the configuration—patient load BW_2_ = 820 [N] and the material parameters of UHMP-PE used inside the body for 8 years, it can be indicated that the main strain in the direction of the vertical axis is 22% smaller than for the base material and is ε_z2_ = 0.30216.

**Figure 23:**
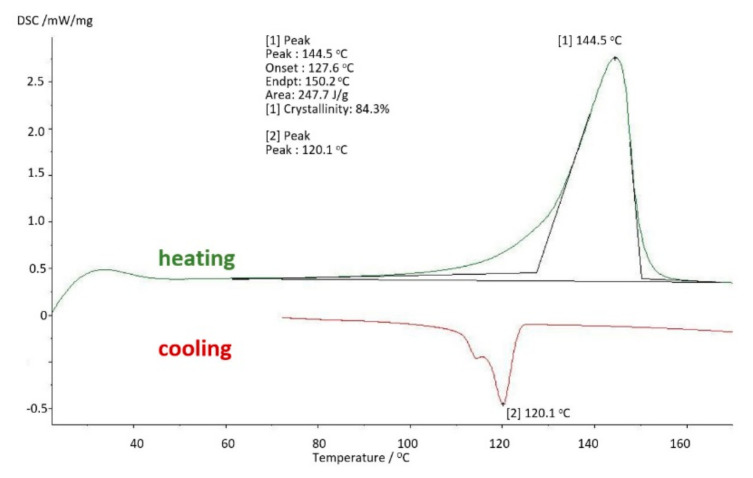
DSC thermogram for polyethylene used in the body for 10 years.

**Figure 24:**
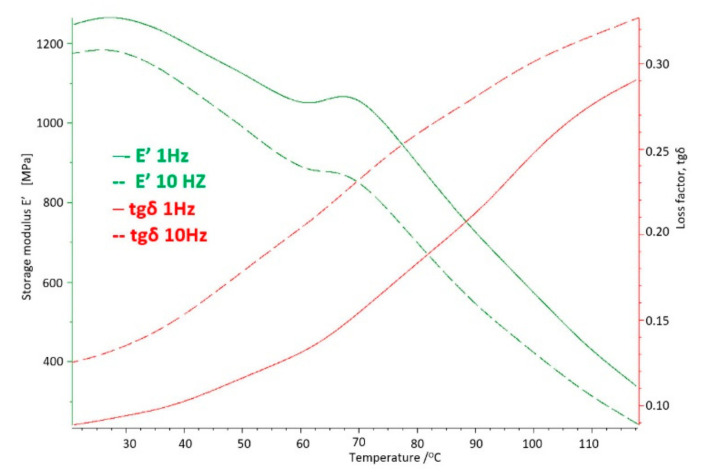
DMTA thermogram for polyethylene used in the body for 10 years.

The samples removed from the human body after 10 years of use were characterised by high surface degradation. In the area of maximum loads, a significant change in the shape of the surface was also visible, indicating cold creep of the material. Figure 23 and Figure 24 below show the results of DMTA and DSC studies of a sample removed from the human body after 10 years of use. On the basis of the conducted tests, it is possible to observe a progressive course of changes in strength parameters and an increase in the stiffness of the material. At this stage of usage, it can be concluded that one of the basic functions of the polyethylene cup, which is vibration damping, has ceased to exist. The amount of the crystalline phase reached K = 84.3%, the material became brittle, cracks and numerous surface defects of UHMW-PE appeared. The melting enthalpy reached the value Q = 247.7 [J/g].

The analysed strength parameters, i.e., the storage modulus is more than twice as high as for the base sample and amounts to E′ = 1101 [MPa] and the mechanical loss coefficient equals tgδ = 0.15.

**Figure 25:**
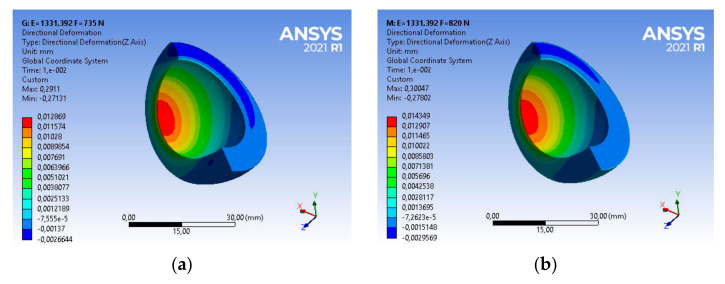
Strains distribution of the acetabular used in the body for 10 years during patient usage: (**a**) BW_1_ = 735 [N], (**b**) BW_2_ = 820 [N].

For a material with strength parameters developed on the basis of DMTA and DSC tests, the strains state of the polyethylene cup is presented in accordance with Figure 25.

**Figure 26:**
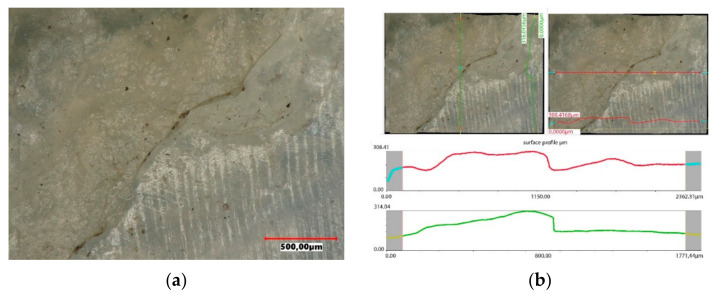
Surface profile of the acetabular used in the body for 10 years: (**a**) surface of the cup, (**b**) surface topography.

The exploitation of the artificial joint for more than 10 years resulted in ridging and the formation of grooves and deep scratches on the implant surface and the separation of fragments of the UHMW-PE surface layer, which is characteristic of fatigue wear of the surface. The UHMW-PE surface topography is characterised by a significant plastic strain, a change in the shape of the contact surface and the presence of numerous wear products associated with the base surface, as shown in Figure 26. Areas with numerous cracks in the layer and layer build-ups, as well as numerous cracks into the base material, were recorded. The wear of the support layer is very high, which leads to an increase in the migration of wear products into the body and a perceived discomfort and pain not only during usage, but also at rest.

## 5. Discussion

Numerical analysis confirmed that with the increase of the strength parameters, the elastic strains of the acetabular decrease. The material has become more rigid, and thus it can be concluded that during loading, the frictional resistance and vibrations transferred from the friction node to the elements fixing the acetabulum in the bone (bone cement in the analysed case) increase. The nature of deformations has not changed significantly, however, their values are disproportionately lower than in the original period of use and amount to respectively ε_z1_ = 0.2911 and ε_z2_ = 0.30047, which is about a 23% decrease in the deformation value compared to the base sample. Table 2 presents a summary of the values of changes in the storage module E′ and the mechanical loss coefficient at a temperature similar to the conditions of use in the human body, i.e., 40 °C in relation to the results of structural tests using the DSC method, including the degree of crystallinity, enthalpy and melting point.

On the basis of the research, the H1 hypothesis was rejected, in favor of the alternative hypothesis H2: The period and intensity of using polyethylene cups inside the human body affects the changes in the strength parameters of UHMW-PE.

The results of the tests obtained by the DMA method at the temperature of 40 °C show changes in the value of the storage modulus E′ corresponding to the stiffness of the material, which significantly increases in the tested range with the time of use compared to the base sample. It is indicated that the material strengthens over time, increasing stiffness.

However, the combination of an aggressive environment and dynamically changing loads that occur during normal use of the prosthesis by patients may result in the formation of scratches and cracks into which the fluids present in the hip joint penetrate and intensify the chemical and mechanical degradation.

The above processes confirm the results of DMA tests, where a significant increase in the E′ storage modulus can be observed from about 528 [MPa] for the base sample, to 1101 [MPa] for the longest-used sample. In the case of the tgδ mechanical loss factor, the greatest differences were noted in the original period of use, which means that this is where the most intense cold creep of polyethylene occurs, The difference of these values is not as large as the storage modulus, which reflects that the damping properties of the tested material have not decreased as significantly as in the case of the storage modulus and enthalpy and the melting point, which proves the participation of the crystalline and amorphous phases in UHMW-PE.

When analysing the results of structural tests by the DSC thermal analysis method, it is also possible to observe a certain convergence with the results of DMA tests. Namely, with each successive time of use and loading of the test sample, the melting enthalpy increases, i.e., the amount of heat in [J] that has been absorbed by the mass of the sample expressed in grams. During the tests, its value increased from 155.3 [J/g] for the base sample to 226.7 [J/g] for the sample removed from the patient’s body at the latest. Along with the change of the enthalpy of melting, the accompanying increase in the amount of the crystalline phase, determined by the value of the degree of crystallinity K, expressed as a percentage, is observed. As mentioned, this process is influenced by cyclical dynamic loads and accompanying phenomena, such as e.g., friction. The greatest changes take place on the contact surface of the acetabular with the metal head and just below the surface of the cup, where a large influence of the mechanical impact of the friction node is observed. The melting point shifts slightly towards higher values and reaches 4–8 °C higher value than the base sample (140.5 °C).

The aetiology of changes should be treated as a set of mechanical and chemical factors, because it is impossible to clearly distinguish which determinant and at what stage of use has a dominant influence. UHMW-PE polyethylene is a material for which the basic structural element is a fibril consisting of alternating crystalline and amorphous regions. The proportions of these phases affect the strength and performance parameters of PE. Cyclic dynamic loads in combination with the aggressive environment of body fluids cause a change in the proportion of the crystalline and amorphous phases, which was verified by empirical research using the DSC method. This translates explicitly into a change in the strength parameters of PE, which in the initial stage of exploitation is an elastic material with very good sliding and vibration-damping properties. After a certain period of use, it loses its elasticity, becomes hard and brittle, and consequently breaks easily. On the basis of the analysis conducted, it can be observed that the degradation process is also influenced by the formation of loose particles and radicals on the surface, which in the process of usage are “pressed” into the PE surface layer, significantly changing the functional properties of the entire component. Artifacts and microcracks caused some of the samples to be damaged (cracked) during the DMTA tests, making it impossible to determine the full course of changes in the entire temperature spectrum. Analysis of surfaces of endoprostheses removed from the human body reveals after some time the degradation of the surface progresses, which results in a change in the topography of the surfaces.

Combined with cyclic loads, the mechanical wear consisting in separating particles from the surfaces in contact led to a change in the separation of wear products and the formation of micro unevenness and loose wear products. The movement of the artificial joint surface caused furrows and deep scratches on the surface of the implant as evidence of fatigue wear of the surface of the implant.

Plastic deformation, a change in the shape of the contact surface, and generation of wear products were observed in acetabulum used to make UHMW-PE. Under cyclic loads, the strength parameters of polyethylene change significantly and the metal particles anchored in them cause a significant damage to joint surfaces.

## 6. Conclusions

The presented results clearly prove that along with the extension of the lifetime of PE, its strength parameters change significantly, which makes it a hard and brittle material that, in turn, contributes to its high wear. Thus, it can be clearly indicated that the use of polyethylene cups is justified, while taking into account the individual anthropometric and goniometric characteristics of the patient. The optimal time of use should not exceed 8 years, and the lower the patient’s weight, the slower the degradation processes. In the case of people with a high body weight, as well as young people (in whom the time of use is assumed to be very long), it is not advisable to use polyethylene cups.

## Figures and Tables

**Table 1:** The number of arthroplasty performed in 2020, broken down by voivodships in Poland [29].

Voivodeship in Poland	Number of Hip Arthroplasty in 2020	Total Number of Arthroplasty in 2020
Lower Silesia	3557	5368
Kuyavian-Pomeranian Voivodeship	2259	3395
Lublin	2286	3288
Lubuskie	1365	2179
Lodzkie	3269	5212
Lesser Poland	4390	7131
Masovian Voivodeship	6771	10,599
Opole Province	1147	1715
Podkarpackie Province	2527	3840
Podlasie	1147	1902
Pomeranian	2458	3749
Silesian	4502	6930
Świętokrzyskie Province	1587	2549
Warmia-Masuria Province	1786	2764
Greater Poland	4816	7670
West Pomeranian	2391	3736

**Table 2:** Summary of E′, tgδ values, temperature and enthalpy of melting, and the proportion of the crystalline phase.

Time of Use in the Body	DMTA Studies		DSC Studies		
	E′ 1 Hz 40 °C, [MPa]	tgδ 1 Hz 40 °C	Melting Point, T_m_ °C	Enthalpy of Melting, [J/g]	Degree of Crystallinity, K%
Base sample	528	0.13	140.8	155.3	53
4 years	557	0.16	148.1	192.5	65.53
8 years	1164	0.17	146.5	232.2	79.18
10 years	1101	0.15	144.5	247.7	84.3

## Data Availability

Data sharing is not applicable to this article.
